# Effect of oral administration of microcin Y on growth performance, intestinal barrier function and gut microbiota of chicks challenged with *Salmonella* Pullorum

**DOI:** 10.1186/s13567-024-01321-x

**Published:** 2024-05-22

**Authors:** Wenjing Li, Zhiwei Zeng, Di Zhou, Guyao Wang, Zepeng Wang, Yu Li, Yu Han, Miaomiao Qin, Changqi Luo, Saixiang Feng, Weisheng Cao

**Affiliations:** 1 College of Veterinary Medicine, South China Agricultural University, Guangzhou, China; 2 Key Laboratory of Zoonosis Prevention and Control of Guangdong Province, South China Agricultural University, Guangzhou, China; 3 Key Laboratory of Zoonosis of Ministry of Agriculture and Rural Affairs, South China Agricultural University, Guangzhou, China; 4 Key Laboratory of Veterinary Vaccine Innovation of the Ministry of Agriculture and Rural Affairs, South China Agricultural University, Guangzhou, China; 5 National and Regional Joint Engineering Laboratory for Medicament of Zoonosis Prevention and Control, South China Agricultural University, Guangzhou, China

**Keywords:** Microcin Y, *Salmonella* Pullorum, intestinal barrier, gut microbiota

## Abstract

The lasso peptide microcin Y (MccY) effectively inhibits various serotypes of *Salmonella* in vitro, but the antibacterial effect against *S*. Pullorum in poultry is still unclear. This study was the first to evaluate the safety and anti-*S*. Pullorum infection of MccY in specific pathogen-free (SPF) chicks. The safety test showed that the body weight, IgA and IgM levels of serum, and cecal microbiota structure of 3 groups of chicks orally administrated with different doses of MccY (5 mg/kg, 10 mg/kg, 20 mg/kg) for 14 days were not significantly different from those of the control group. Then, the chicks were randomized into 3 groups for the experiment of anti-*S*. Pullorum infection: (I) negative control group (NC), (II) *S*. Pullorum-challenged group (SP, 5 × 10^8^ CFU/bird), (III) MccY-treated group (MccY, 20 mg/kg). The results indicated that compared to the SP group, treatment of MccY increased body weight and average daily gain (*P* < 0.05), reduced *S*. Pullorum burden in feces, liver, and cecum (*P* < 0.05), enhanced the thymus, and decreased the spleen and liver index (*P* < 0.05). Additionally, MccY increased the jejunal villus height, lowered the jejunal and ileal crypt depth (*P* < 0.05), and upregulated the expression of *IL-4*, *IL-10*, *ZO-1* in the jejunum and ileum, as well as *CLDN-1* in the jejunum (*P* < 0.05) compared to the SP group. Furthermore, MccY increased probiotic flora (*Barnesiella*, etc.), while decreasing (*P* < 0.05) the relative abundance of pathogenic flora (*Escherichia* and *Salmonella*, etc.) compared to the SP group.

**Supplementary Information:**

The online version contains supplementary material available at 10.1186/s13567-024-01321-x.

## Introduction

Pullorum disease (PD), caused by *Salmonella* Pullorum (*S*. Pullorum) infection, has caused grievous economic harm to the poultry industry in developing countries [1]. *S*. Pullorum infection induces systemic infection and white diarrhea symptoms, resulting in extremely high rates of morbidity and mortality among chicks aged 2–3 weeks. Some *S*. Pullorum-tolerant chicks become long-term carriers and eventually develop into subclinical and persistent infections, causing horizontal and vertical transmission with a negative economic impact on the poultry industry [2]. In recent years, epidemiological surveys in China have identified *S*. Pullorum as the most prevalent *Salmonella* serotype on large-scale chicken farms [3, 4]. Antibiotics, including beta-lactams, aminoglycosides, and quinolones, have a historical application in preventing and treating *Salmonella* infections. However, the widespread use of antibiotics in the poultry industry for growth promotion and treating bacterial infections has led to the evolution of antibiotic-resistant pathogens and the challenge of drug residues [5–7]. “Antibiotic-free” feed has become an inevitable trend in the development of the international livestock industry, with many countries restricting the use of antibiotics [8, 9]. Therefore, the development of safe and effective alternatives to antibiotics to prevent bacterial infections and reduce the incidence rate of intestinal diseases in poultry is one of the critical issues that need to be urgently addressed in animal husbandry.

Microcin are low-molecular-weight, ribosomally synthesized, bacterial-inhibitory peptides that provide a competitive advantage among *Enterobacteriaceae* in the gut [10]. They have been shown to selectively remove pathogenic bacteria from the gastrointestinal tract and inhibit the growth of intestinal pathogens [11]. Microcin J25 (MccJ25) effectively reduced intestinal *Streptococcus* colonization, and enhanced growth capability and gut health in poultry and weaned piglets [12]. Additionally, MccJ25 markedly downregulated the serum levels of proinflammatory cytokines in chicks infected with *Escherichia coli* (*E*. *coli*) and *Salmonella* [13]. In the mouse gut, microcin I47 (MccI47) reduces the colonization of carbapenem-resistant *Klebsiella pneumoniae* [14]. These findings suggest that microcin has the potential to improve animal performance while reducing intestinal pathogen development.

Microcin Y (MccY), encoded by a gene cluster isolated from *Salmonella* Enteritidis, is a class I microcin containing 21 amino acids with strong antimicrobial action against *Salmonella* and *Shigella* [15]. The recognition and absorption of MccY involves the outer membrane protein FhuA, the inner membrane protein SbmA, and the Ton complex [15, 16]. One of these, the structural variation of FhuA, caused the differences in the biological antibacterial activity between MccY and MccJ25, and as a result, MccY significantly expanded the antimicrobial spectrum of MccJ25 against *Salmonella* [15]. Our previous research showed that MccY regulates intestinal barrier function and gut microbiota to attenuate *S*. Typhimurium-induced intestinal inflammation in mice [17]. However, studies utilizing the protective effects of MccY to reduce *Salmonella* infection in chickens have not been reported.

In this study, we hypothesized that MccY might have a beneficial effect on *S*. Pullorum infection in chicks; therefore, the current study was devoted to investigating the regulatory effects of oral administration of MccY on the growth performance, intestinal barrier function, and gut microbiota of chicks challenged with *S*. Pullorum.

## Materials and methods

### Bacterial strains, plasmids, and primers

All strains, plasmids and primers used in this study can be found in Additional files 1, 2 and 3, respectively.

### Preparation of MccY

MccY was produced by *E*. *coli* (YL01) and MccJ25 was produced by *E*. *coli* (YL02) after 24 h of incubation in M9 medium with 30 μg/mL kanamycin and 0.5 mM IPTG. Next, following centrifugation, MccY or MccJ25 from the supernatant was enriched by reversed-phase solid-phase extraction (SPE), and then purified and quantified by LC-QQQ-MS as described previously [15, 17]. MccY and MccJ25 were finally obtained by lyophilization and resuspension at a concentration of 5 mg/mL.

### Antimicrobial activity test

As described in our previous study [16], a single colony of *S*. Pullorum was selected and cultured in LB broth at 37 °C and 200 rpm until OD600 = 0.8. The culture was added to LB medium containing 0.5% agar at a ratio of 1:1000 to prepare plates. Next, 50 μL of 2 mg/mL MccY was spotted on the plate surface, or MccY was continuously diluted from 250 μM to 0.5 μM, and subsequently, 50 μL of the diluted solution was sequentially spotted on the plate surface. After allowing for drying, the plates were cultured at 37 °C for 24 h to observe the antibacterial zones as an activity indicator. All experiments were performed in triplicate.

### MIC assay

The minimum inhibitory concentration (MIC) of MccY was determined using a 96-well plate as described in our previous study [15]. Strains were cultured overnight on MH agar plates, then a single colony was inoculated in MH broth and cultivated at 200 rpm and 37 ℃ to OD600 = 0.8. After that, 50 μL culture was diluted 1000-fold to the 96-well plate. Then, 50 μL of different concentrations of MccY from 250 μM to 0.0125 μM were added into the previous culture successively. The mixed cultures were co-incubated at 37 ℃ for 24 h, and the MIC values were determined by measuring A_600_. All experiments were repeated three times.

### Preparation of *S*. Pullorum-challenged solution

A single colony of *S*. Pullorum was cultured in LB broth at 200 rpm and 37 ℃ for 6–8 h until it reached the logarithmic phase of growth. After centrifuging the culture liquid at 5000 rpm and 4 ℃ for 10 min and removing the supernatant, the cells were washed thrice with PBS and suspended in PBS until the final concentration reached 1 × 10^9^ CFU/mL.

### Model design of Pullorum disease

All of the specific pathogen-free (SPF) White Leghorn chicks were acquired from Xinxing DahuaNong Poultry Egg Co., Ltd., raised in cages with free access to feed and water. A total of 45 one-day-old SPF chicks with similar body weight (BW) were randomly divided into three groups, each consisting of 15 chickens. At day 3 and day 5, the three groups were orally challenged with 0.5 mL (1 × 10^9^ CFU/mL) of different bacterial solutions (*S*. Pullorum 284, 429 and CVCC 526), respectively. The growth of chicks was observed every day, and the fecal bacterial load was continuously monitored on 1, 3, 5, 7 and 14 days post-infection (dpi). Chicks were euthanized on dpi 3, 7 and 14, and liver and cecum samples were collected and counted for bacterial load.

### Safety evaluation of MccY in chicks

A total of 40 one-day-old SPF chicks were randomized into four groups (control group, 5 mg/kg MccY group, 10 mg/kg MccY group and 20 mg/kg MccY group, *n* = 10). The four groups of chicks were orally administered MccY diluted in phosphate-buffered saline (PBS) at 5 mg/kg, 10 mg/kg, 20 mg/kg and an equal volume of PBS from day 5 for 14 days continuously. The growth of chickens was observed daily. After 14 days of giving MccY (day 19), the body weight of the chickens was measured, and anticoagulant blood was collected through the jugular vein. The chickens were dissected and four chickens were randomly selected to collect the cecal contents.

### Experimental design of treating *S*. Pullorum- infected chicks with MccY

A total of 90 one-day-old SPF were randomized into three treatment groups with five replicates of six chickens per replicate. The experimental treatments included a negative control group (NC, neither *S*. Pullorum infection nor treatment), a positive control group (SP, *S*. Pullorum infection but without treatment) and an MccY-treated group (MccY, *S*. Pullorum infection but with MccY treatment).

**Figure 1:**
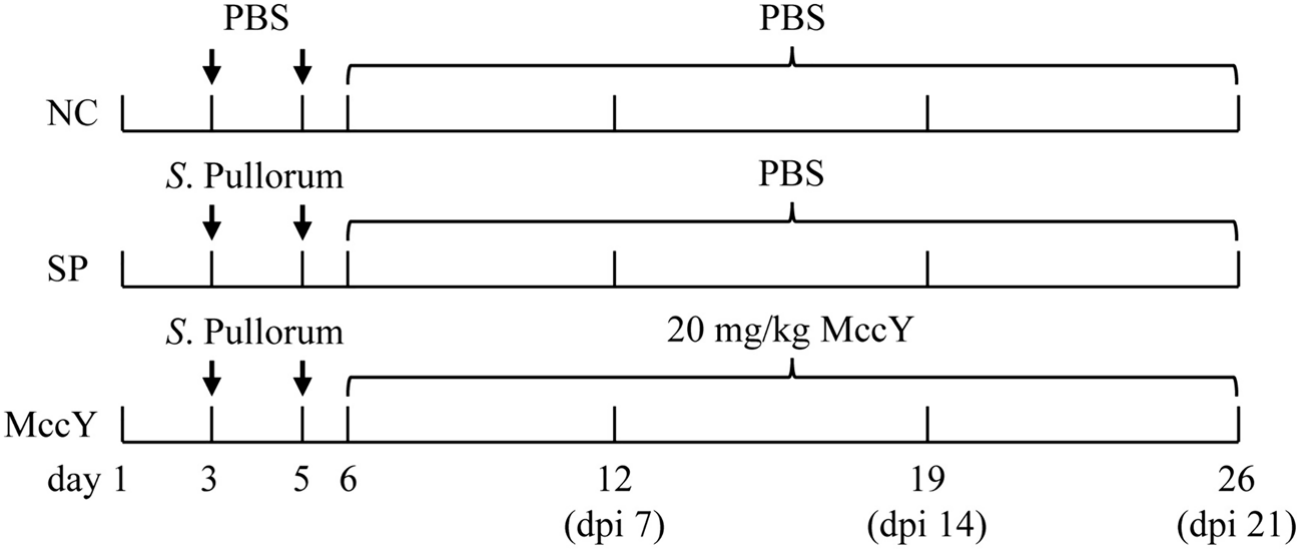
**Experimental design of MccY treatment in chicks infected with *S.*** **Pullorum.** dpi: days post-infection. NC: negative control group, SP: positive control group, MccY: MccY-treated group.

At days 3 and 5, chicks in the SP, and MccY groups were orally challenged with 0.5 mL (1 × 10^9^ CFU/mL) of *S*. Pullorum solution, while chicks in the NC group were orally administered the same volume of PBS (Figure 1). On dpi 1, chicks in MccY were treated with 20 mg/kg MccY diluted in PBS, while chicks in NC and SP were given the same volume of PBS.

### Sample collection

Body weight was weighed for each chick to calculate its average daily gain (ADG). Feces, liver and cecum samples were collected from each group for *Salmonella* enumeration. Blood samples were collected jugular vein and centrifuged at 3000 rpm and 4 °C for 5 min, and then the serum was collected and stored at −80 °C until analysis. After euthanasia for cervical dislocation, we weighed and recorded the immune organs, including the thymus, spleen, bursa of Fabricius and liver, and finally calculated the immune organ index. Two middle segments of the jejunum and ileum (approximately 1 cm in length) were cut separately, and one segment was rinsed with PBS and then fixed in 4% (w/vol) paraformaldehyde solution for further morphological analysis. The other jejunal and ileal samples were placed into sterile tubes, rapidly snap-frozen in liquid nitrogen, and kept at −80 °C until mRNA expression was determined. The cecum contents (approximately 500 mg) of four chicks per group were gathered, flash-frozen in liquid nitrogen, and preserved at −80 °C for the bioinformatic analysis of the gut microbiota. Cecum content (approximately 500 mg) samples were collected for *Salmonella* load counting.

### Cytokine production measurement

The IgA and IgM of serum from the different groups were determined by using ELISA kits (Enzyme-linked Biotechnology, Shanghai, China) according to the manufacturer’s instructions, and the concentrations were calculated from standard curves.

### Determination of* S*. Pullorum load in feces, liver and cecum

Similar to the previous study on MccJ25 [18], feces, liver and cecum samples (500 mg) were homogenized with 5 mL of sterile PBS in tubes, diluted to 10^−1^, 10^−2^, 10^−3^, 10^−4^, 10^−5^, and 100 µL of each dilution, evenly spread on 1.5% XLT-4 agar plates and cultured at 37 °C for 48 h to count colonies. All counts were repeated in triplicate.

### Morphological analysis of the jejunum and ileum

The paraformaldehyde-fixed intestinal samples were dehydrated, embedded in paraffin, sectioned about 5 µm and then stained with hematoxylin and eosin (HE) for histological analysis. After scanning the stained sections with the Pannoramic DESK pathology scanner, the villus height (VH) and crypt depth (CD) were measured at 100× magnification using Slide Viewer software, and then the ratio of villus height to crypt depth (V/C) was computed. Five intact villi from each section were selected for five repeated measurements to calculate the average value.

### RNA-Seq analysis of the jejunum and ileum

Following the instructions, total RNA (50 mg) from the intestinal tissues was extracted using RNAiso Reagent (Fast Gene, Shanghai, China), and the purity and concentration of total RNA were determined at 260 and 280 nm, respectively. Then, cDNA was constructed using HiScript II Q RT SuperMix for qPCR (+ gDNA wiper) (Vazyme Biotech Co., Ltd, Nanjing, China). The expression of target genes, including interleukin-4 (*IL-4*), *IL-6*, *IL-10*, interferon-γ (*IFN-γ*), tumor necrosis factor-α (*TNF-α*), zonula occludin-1 (*ZO-1*), Occludin (*OCLN*), Claudin-1 (*CLDN-1*) and Mucin-2 (*MUC-2*) was measured by using quantitative real-time PCR (qPCR) reactions in duplicate performed with 2 × Real Star Fast SYBR qPCR Mix (Genstar, Beijing, China) in the Bio-Rad-CFX Real-Time PCR system (Bio-Rad, USA). The *GAPDH* gene served as an internal control in this study, and Additional file 3 displays the sequences of gene primers synthesized by Sangon Biotech (Shanghai, China). The 2^−ΔΔCt^ method was utilized to standardize the relative expression level of target genes, and all tests were performed in triplicate.

### 16S ribosomal RNA gene sequencing of the cecal microbiome

Based on previous studies, microbial metagenomic DNA was extracted from cecal contents (approximately 200 mg of each sample), and then the concentration, purity and integrity were detected [19]. The 16S rRNA genes V3 to V4 region were amplified by PCR using primers 338F and 806R [20], and then the PCR amplicons were purified, quantified and sequenced on the Illumina NovaSeq platform using the NovaSeq 6000 SP Reagent Kit (Personal Biotechnology Co., Ltd., China). Microbiome bioinformatics analyses, including α-diversity indices [21, 22], and species composition analysis were performed with QIIME2 2019.4 and R packages (v3.2.0) [23].

### Statistical analysis

Dates were detected using one-way analysis of variance (ANOVA) in GraphPad Prism 9.0 and SPSS 20.0. Differences were considered significant at *P* < 0.05 or extremely significant at *P* < 0.01. Data are presented as the mean ± SEM.

## Results

### Antibacterial activity of MccY in vitro

**Figure 2:**
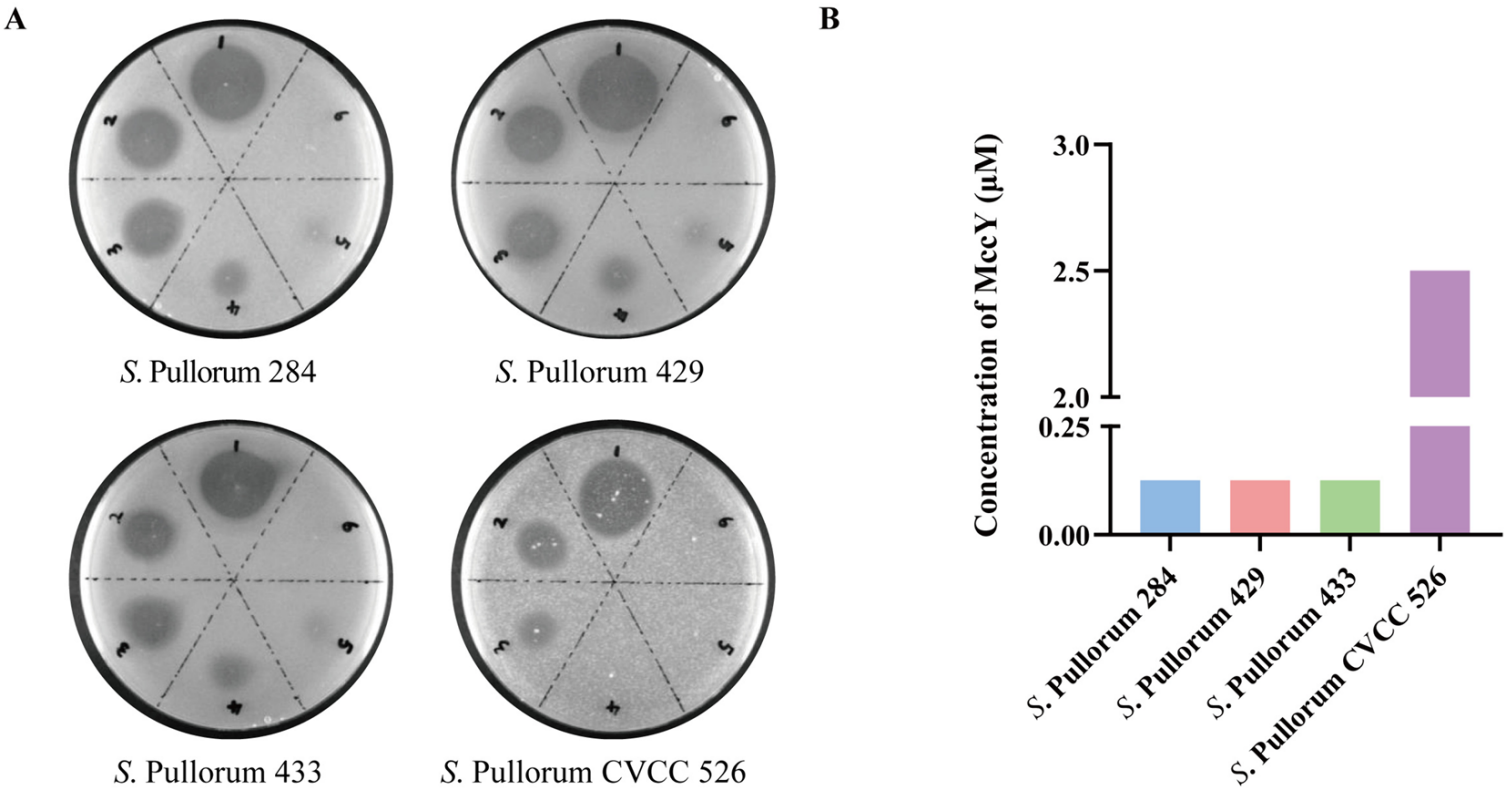
**Sensitivity of *S.*** **Pullorum to MccY.** **A** Spot-on-lawn assay of MccY against *S*. Pullorum isolates 284, 429, 433 and CVCC 526. Serial dilutions from 250 μM (spot 1) to 0.5 μM (spot 6) were tested. **B** MIC assays of *S*. *Pullorum*.

In this study, 3 strains of *S*. Pullorum isolated from diseased chickens and 1 standard strain were selected for the bacterial inhibition test of MccY in vitro. As shown in Additional file 4, *S*. Pullorum 284, 429, 433 and CVCC 526 were exhibited sensitivity to MccY, but resistant to MccJ25. Additionally, the spot-on-lawn results (Figure 2A) visually demonstrated the difference in sensitivity of *S*. Pullorum strains towards MccY. Simultaneously, the MIC assays (Figure 2B) revealed that the MIC values for isolates 284, 429, and 433 were determined as 0.125 μM, and that of CVCC 526 reached 2.5 μM. The above results showed that both clinical isolates and standard strain were highly sensitive to MccY, thereby supporting its potential use for establishing a model of Pullorum disease.

### Model of Pullorum disease

3 strains of *S*. Pullorum were used to induce infection in chickens, and the bacterial loads in feces and organs were monitored to identify a suitable model of Pullorum disease. On dpi 1, chicks challenged *S*. Pullorum 284 exhibited lethargy without signs of white diarrhea; whereas chicks challenged *S*. Pullorum 429 displayed both mental depression and a 100% incidence of white diarrhea. Additionally, partial chicks infected with CVCC 526 developed white diarrhea. These observations were consistent with the results on fecal bacterial load presented in Additional file 5. The fecal *Salmonella* load of *S*. Pullorum 429 was monitored up to 7 days after the challenge, whereas those of CVCC 526 could only be maintained for 3 days after the challenge. Furthermore, only *S*. Pullorum 429 exhibited stable detection in liver and cecum tissues. Based on these findings, *S*. Pullorum 429 is more suitable for establishing a chicken model of Pullorum disease.

### Effect of MccY on chicks

**Figure 3:**
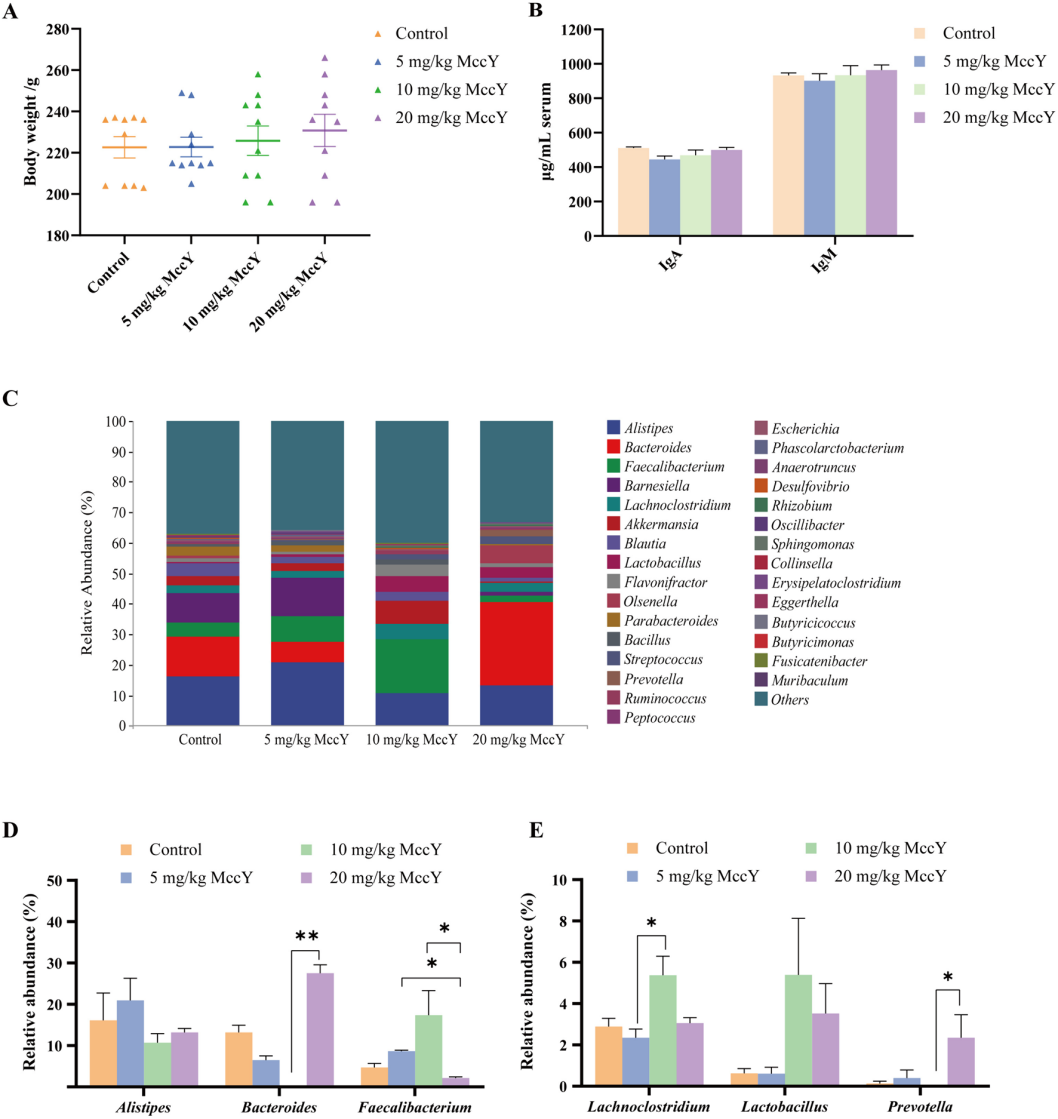
**Effect of oral administration of MccY on chicks.** **A** Body weight (BW) in MccY-given chicks at d 19, *n* = 10. **B** IgA and IgM expression of chick serum at day 19, *n* = 5. **C**–**E** The relative abundance of cecal microbiota at the genus levels at d 19, *n* = 4. Dates were shown as the means ± SEM. *P < 0.05. **P < 0.01.

The safety of MccY on chicks remains uncertain; therefore, this study initially examined the effects of different concentrations of orally administered MccY on body weight, immune factor, and intestinal flora in chicks for 14 consecutive days. As shown in Figure 3A, compared to the control group, chickens given 20 mg/kg MccY tended to increase body weight, but there were no significant differences among the four groups.

The levels of IgA and IgM in chicken serum exhibited no significant alteration after oral administration of low, medium and high concentrations of MccY compared with the control group (Figure 3B), suggesting a low immunogenic response elicited by MccY.

The cecum contents collected on the 14th day of oral MccY were subjected to analysis of intestinal flora. The results revealed that there was a total of 395 operational taxonomic units (OTUs) across the four groups, with the unique OTUs ranked in descending order as follows: 5 mg/kg MccY group, control group, 20 mg/kg MccY group, and 10 mg/kg MccY group (see Additional file 6). The index of Simpson, Shannon, Chao1, and Observed species did not exhibit any significant differences between the four groups (see Additional file 6), suggesting that there was no substantial alteration in the richness and diversity of communities among different doses of MccY-given groups compared to the control group. The relative abundance of the flora at the genus level is depicted in Figure 3C, with *Alistipes*, *Bacteroides*, *Faecalibacterium*, *Barnesiella*, and *Lachnoclostridium* being the top five genera. Compared to the control group, the 5 mg/kg MccY group showed a 3.93% increase in the relative abundance of *Faecalibacterium*; the 10 mg/kg MccY group exhibited an increase in the relative abundance of *Faecalibacterium* by 12.64%, *Lachnoclostridium* by 2.48%, *Lactobacillus* by 4.77% and a decrease in *Bacteroides* by 13.04%; and the 20 mg/kg MccY group showed an increase in the relative abundance of *Lactobacillus* and *Prevotella* by 2.90% and 2.22%. Compared with the 10 mg/kg MccY group, the 20 mg/kg MccY group showed significant increases in the relative abundance of *Bacteroides* (*P* < 0.01) and *Prevotella* (*P* < 0.05), while the relative abundance of *Faecalibacterium* was significantly decreased (*P* < 0.05) (Figures 3D, E).

Oral administration of 20 mg/kg MccY did not exert a significant impact on the body weight and serum immune factor levels of chickens. Despite inducing alterations in the relative abundance of certain beneficial genera, it did not disrupt the normal intestinal microecology. Based on the above results, a dose of 20 mg/kg MccY was administered for the treatment of chickens infected with *S*. Pullorum to ensure optimal therapeutic efficacy.

### Growth performance on chicks challenged with *S*. Pullorum

**Figure 4:**
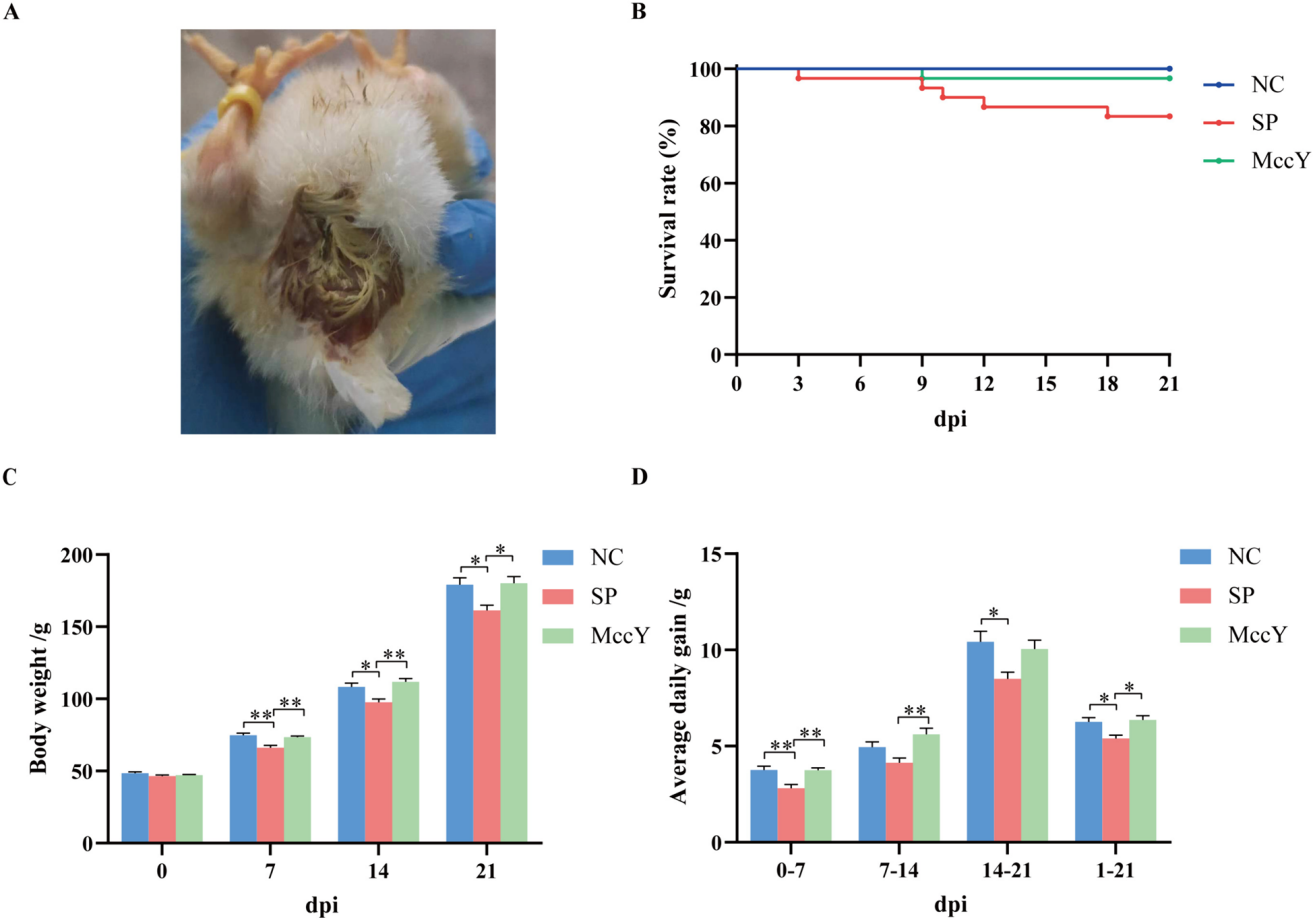
**Effect of MccY on the growth performance of chicks challenged with** ***S.*** **Pullorum.** **A** Symptoms of white diarrhea at dpi 1. **B** Survival curve. **C** Body weight changes in *S*. Pullorum infection. **D** Average daily gain change in *S*. Pullorum infection. Dates were shown as the means ± SEM. *P < 0.05. **P < 0.01. dpi: days post-infection. NC: negative control group, SP: positive control group; MccY: MccY-treated group.

At dpi 1, All chickens except the chicks in the NC group showed symptoms of white diarrhea (Figure 4A). The survival curve (Figure 4B) exhibited that the survival rate of chickens in the SP group was 83.33% (25/30), while it reached 100% (30/30) in the NC group (blank control group), and achieved a rate of 96.67% (29/30) in the MccY group, indicating that administration of MccY could improve chicken survival. The effects of different experimental groups on BW and ADG are shown in Figures 4C, D. Compared to the NC group, the chicks in the SP group showed a significant decrease in the BW (*P* < 0.05), while no statistical differences were seen in the BW of the MccY groups at dpi 7, 14 and 21. In addition, the ADG of the chicks in the SP group was lower than that of the chicks in the NC group (*P* < 0.05) from dpi 0 to 7, dpi 14 to 21 and dpi 1 to 21, and that of the chicks in the MccY group (*P* < 0.05) from dpi 0 to 7, dpi 7 to 14 and dpi 1 to 21. There was no significant difference in the BW and ADG between the NC and MccY groups during the whole experiment. The above results indicated that oral administration of MccY can alleviate weight loss in chicks infected with *S*. Pullorum.

### Bacterial load

**Figure 5:**
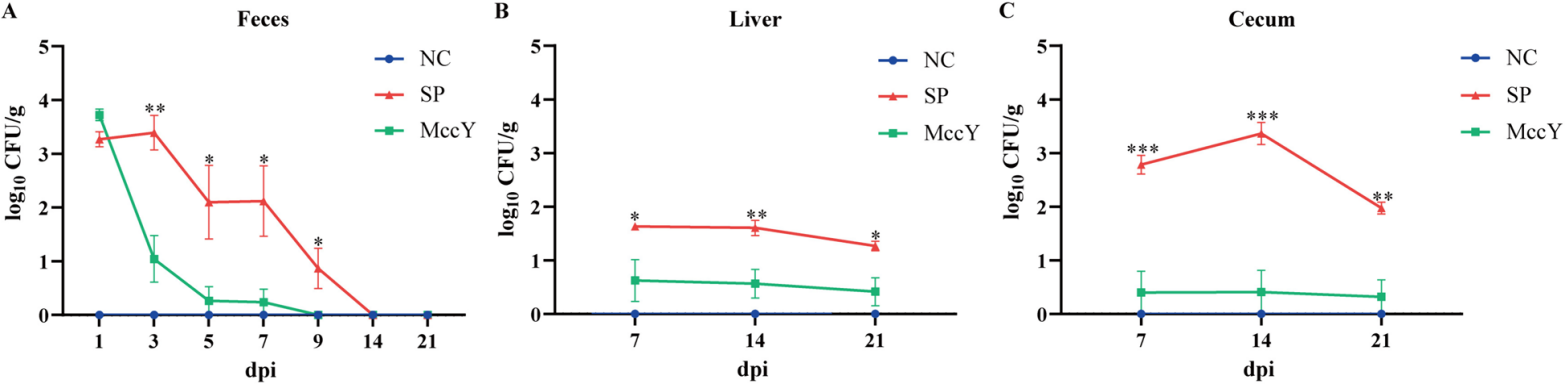
***S. *Pullorum load in feces (A), liver (B) and cecum (C).** Dates were shown as the means ± SEM (*n* = 5), *P < 0.05, **P < 0.01, ***P < 0.001. dpi: days post-infection. NC: negative control group; SP: positive control group; MccY: MccY-treated group.

The *S*. Pullorum burden in feces, liver and cecum was presented in Figure 5. Compared to the SP group, a significant decrease by 99.3%, 99.8%, 99.8% and 91.3% in fecal *S*. Pullorum load was observed in the MccY-treated group at dpi 3, 5, 7 and 9 (*P* < 0.05) (Figure 5A). Moreover, MccY treatment markedly reduced *S*. Pullorum load in the liver by 61.3%, 85.8% and 76.0% compared with the SP group, respectively (*P* < 0.05) (Figure 5B) at dpi 7, 14, and 21. Additionally, at dpi 7, 14, and 21, MccY treatment resulted in a substantial decrease of *S*. Pullorum levels in the cecum by 97.4%, 99.4%, and 92.7% correspondingly (*P* < 0.01) (Figure 5C). The administration of MccY resulted in a 70% reduction in *Salmonella* colonization in the liver and a more than 90% decrease in *Salmonella* colonization in the gut.

### Immune organ index on chicks challenged with *S*. Pullorum

**Figure 6:**
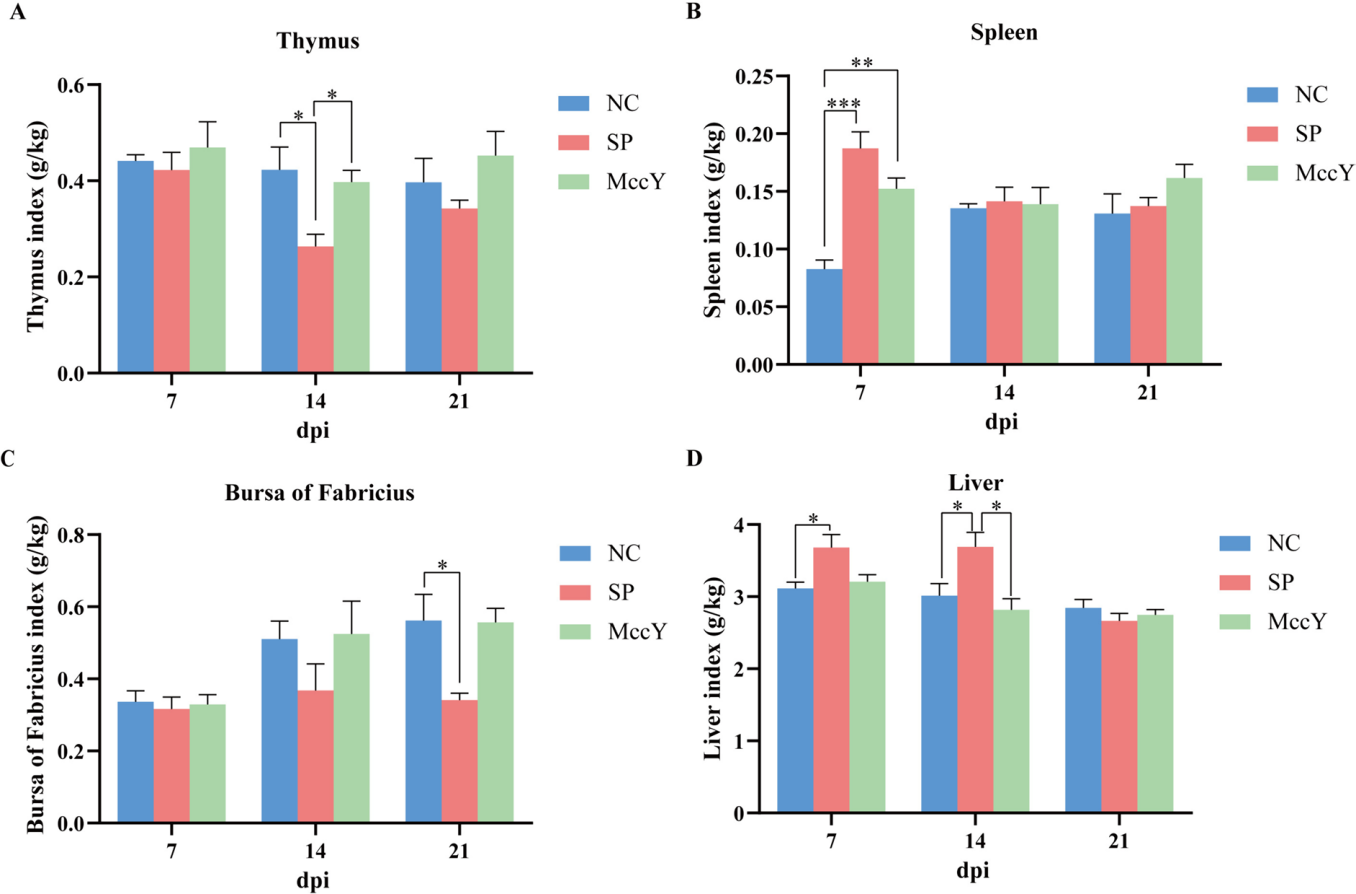
**Effect of MccY on the immune organ index of chicks challenged with** ***S.*** **Pullorum.** **A** Thymus index. **B** Spleen index. **C** Bursa of Fabricius index. **D** Liver index. Dates were shown as the means ± SEM (*n* = 5). *P < 0.05. **P < 0.01. ***P < 0.001. dpi: days post-infection. NC: negative control group; SP: positive control group; MccY: MccY-treated group.

The immune organ indies of the chicks in the three groups were presented in Figure 6. The thymus index of the chicks in the SP group was lower than that of the NC and MccY group at dpi 14 (*P* < 0.05), and with a downward trend at dpi 7 and 21 (Figure 6A). The spleen index of the SP group was markedly higher than that of the NC group (*P* < 0.001) and the MccY group (*P* < 0.01) at dpi 7, whereas no obvious differences were shown among the three groups at dpi 14 and 21 (Figure 6B). The bursa of Fabricius index in the SP group demonstrated a significant decrease compared to the NC group at dpi 21 (*P* < 0.05) (Figure 6C). The liver index of the SP group exhibited an increase compared to the NC group at dpi 7 (*P* < 0.05), and the NC and MccY groups at dpi 14 (*P* < 0.05) (Figure 6D). The above results suggested that MccY treatment alleviates the suppressive effects on the thymus and bursa of Fabricius, as well as the splenomegaly and hepatomegaly induced by *S*. Pullorum infection.

### Intestinal morphology on chicks challenged with *S*. Pullorum

**Figure 7:**
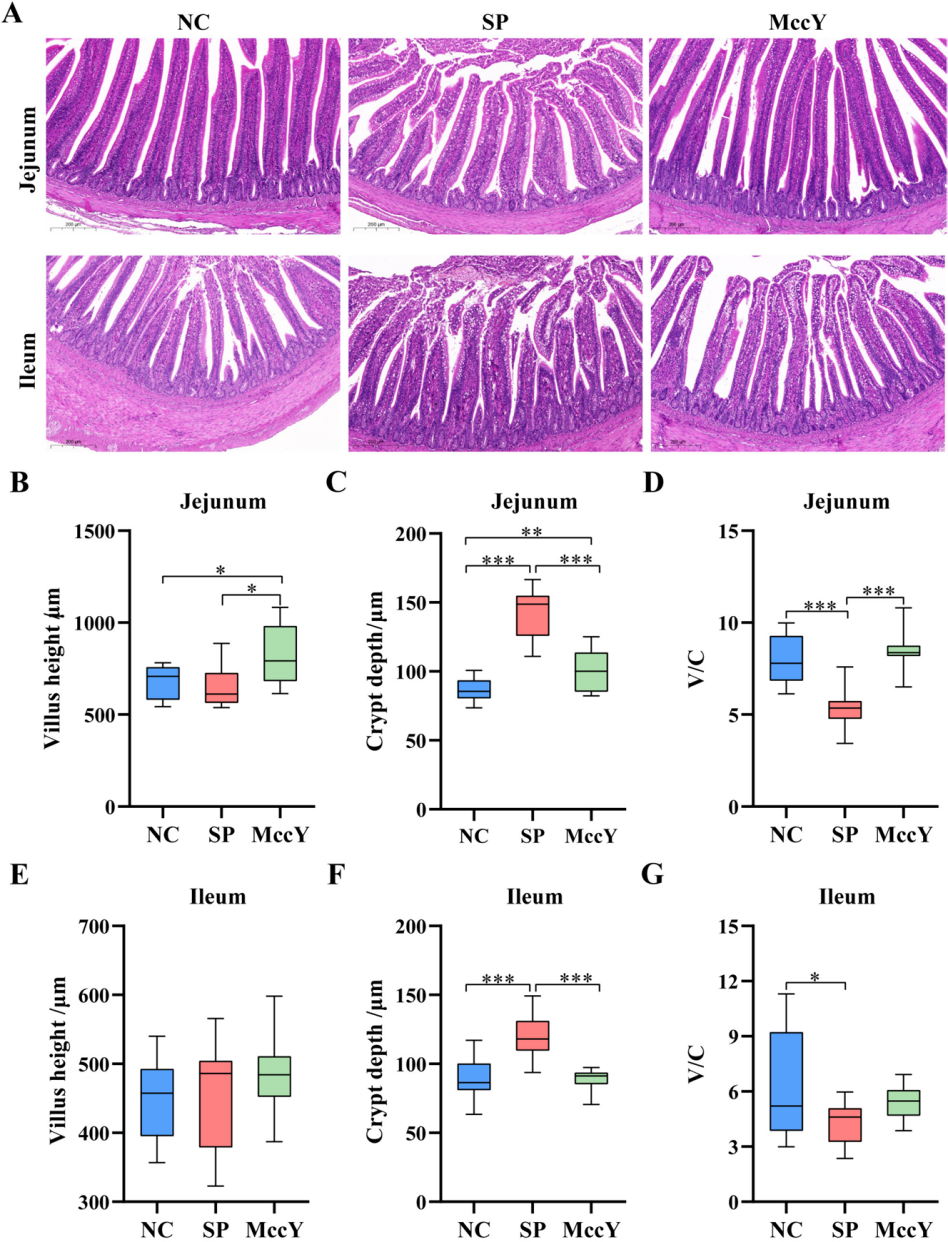
**The morphology of the jejunum and ileum at dpi 21.** **A** Jejunal and ileal morphological structure (scale bar: 200 μm). Jejunal villus height (**B**), crypt depth (**C**) and the ratio of villus height to crypt depth (V/C) (**D**). Ileal villus height (**E**), crypt depth (**F**) and the ratio of villus height to crypt depth (V/C) (**G**). Dates were shown as means ± SD (*n* = 20). *P < 0.05, **P < 0.01, ***P < 0.001. C: negative control group; SP: positive control group; MccY: MccY-treated group.

The jejunal and ileal morphological structures of chicks at dpi 21 are shown in Figure 7A. After *S*. Pullorum infection, inflammatory cell infiltration, uneven crypt arrangement and epithelial cell detachment were observed in the mucosal layers of the jejunum and ileum in the SP group, indicating that *S*. Pullorum infection caused intestinal mucosal damage, but these injuries were not found in the MccY-treated group. On further analysis, compared with the NC group, *S*. Pullorum infection significantly increased the jejunal (Figure 7C) and ileal (Figure 7F) crypt depth (*P* < 0.001), obviously reduced the jejunal (Figure 7D) and ileal (Figure 7G) V/C (*P* < 0.05) t. Interestingly, compared with the SP group, MccY treatment significantly decreased the jejunal (Figure 7C) and ileal (Figure 7F) crypt depth (*P* < 0.01), increased the jejunal villus height (Figure 7B), and jejunal V/C (Figure 7D) (*P* < 0.05). These results showed that the addition of MccY could prevent intestinal villus shedding and improve gut integrity to a certain extent.

### Gene expression in the intestinal mucosa on chicks challenged with *S*. Pullorum

**Figure 8:**
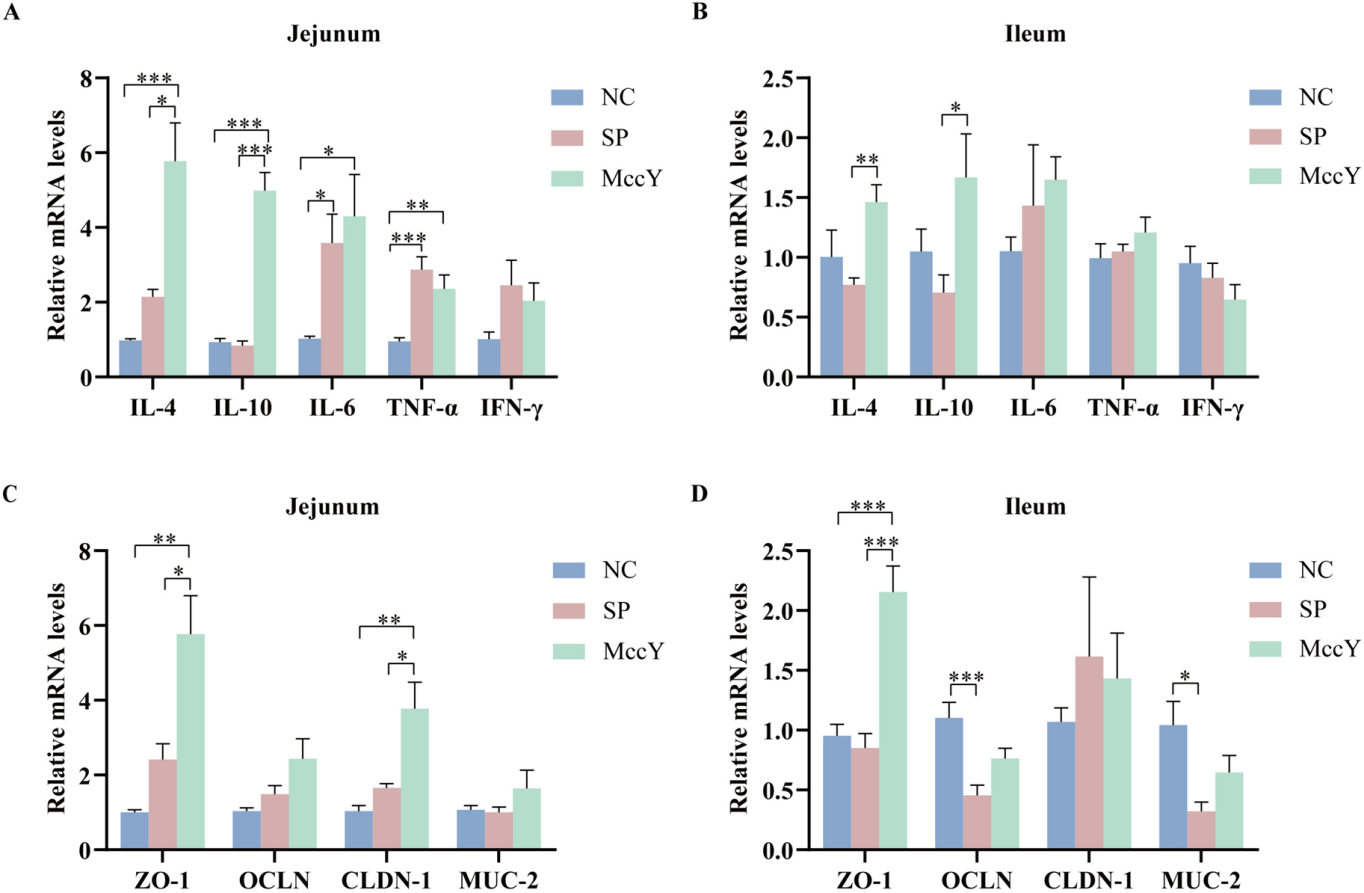
**The mRNA expression of inflammatory factors and tight junction proteins at dpi 21.** Expression of inflammatory factors in the jejunum (**A**) and ileum (**B**). Expression of tight junction proteins in the jejunum (**C**) and ileum (**D**). Dates were shown as the means ± SEM. *P < 0.05, **P < 0.01, ***P < 0.001. NC: negative control group, SP: positive control group, MccY: MccY-treated group.

The mRNA expression levels of the target genes related to the inflammatory factors and tight junction proteins in the jejunal and ileal mucosa of chicks at dpi 21 are shown in Figure 8. Compared to the NC group, the mRNA expressions of pro-inflammatory factors *IL-6* and *TNF-α* in the jejunum of the SP and MccY group infected with *S*. Pullorum were significantly up-regulated (*P* < 0.05) (Figure 8A), and *IFN-γ* displayed an up-regulated trend, indicating that inflammatory responses in chickens infected with *S*. Pullorum. Notably, expressions of the anti-inflammatory factors *IL-4* and *IL-10* in the jejunum of the MccY group exhibited significantly higher levels compared to those observed in the other two groups (*P* < 0.05) (Figure 8A). Additionally, the expressions of *IL-4* and *IL-10* in the ileum of the MccY group were significantly increased when compared to the SP group (Figure 8B), suggesting that MccY regulated intestinal mucosal immunity by secreting more anti-inflammatory factors, thereby improving the chicks’ defenses against *S*. Pullorum infections.

The mRNA expression of gut barrier genes in chicks was further determined. The mRNA expressions of *OCLD* and *MUC-2* in the ileum of the SP group were significantly lower than those of the NC group (*P* < 0.05) (Figure 8D), indicating that *S*. Pullorum infection disrupts the integrity of ileal tight junctions. Furthermore, compared to both the NC and SP groups, oral administration of MccY significantly upregulated *ZO-1* and *CLDN-1* mRNA expressions in the jejunum, as well as *ZO-1* expression in the ileum (*P* < 0.05) (Figures 8C, D), suggesting that MccY enhanced intestinal barrier function by upregulating the mRNA expression of genes related to tight junction proteins.

### Cecal microbiota analysis on chicks challenged with *S*. Pullorum

**Figure 9:**
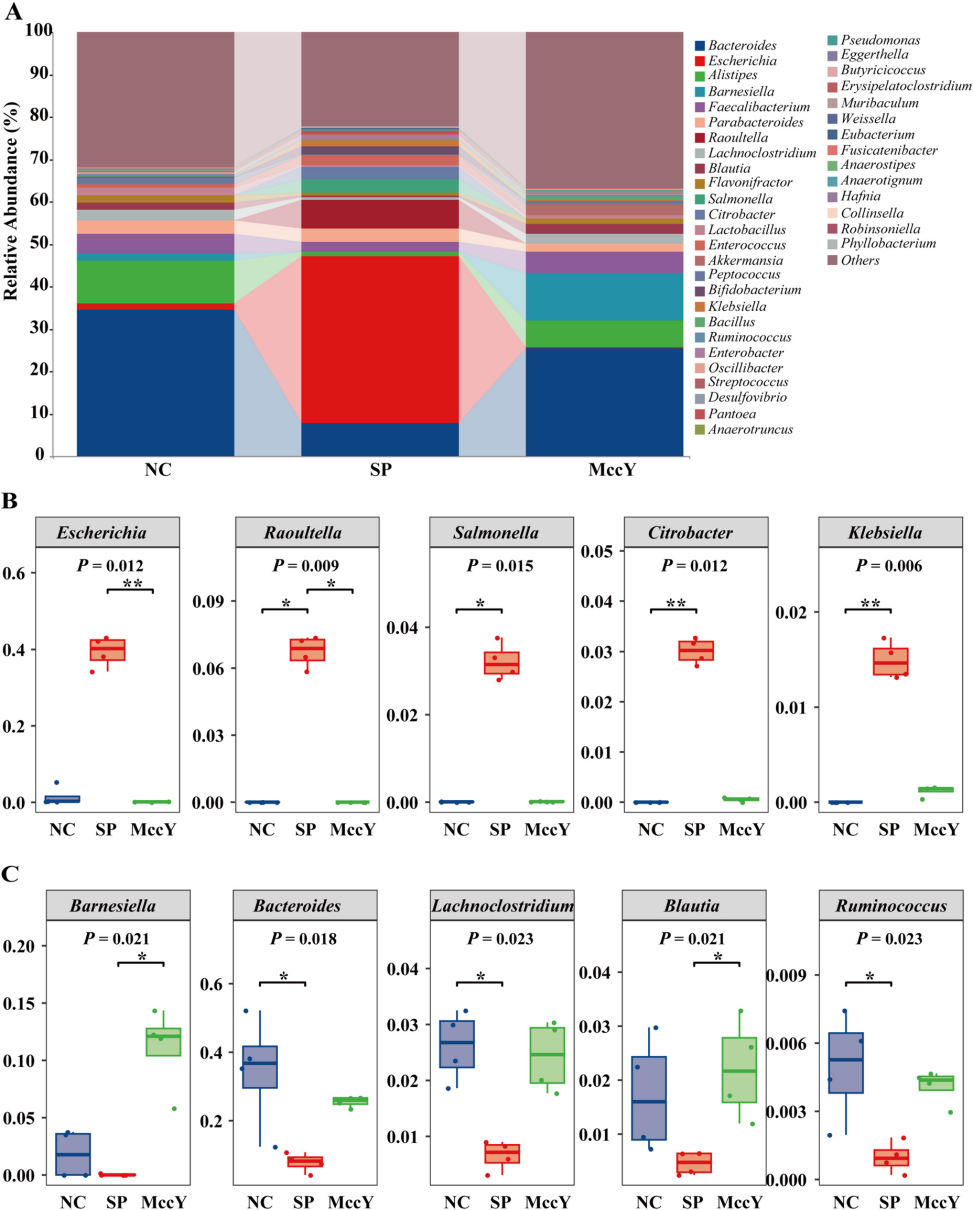
**Effects of MccY treatment on the cecal microbiota structure in chicks infected with *S.*** **Pullorum.** **A** The relative abundance of cecal microbiota of chicks at the genus level at dpi 21. **B** The relative abundance of *Escherichia*, *Raoultella*, *Salmonella*, *Citrobacter*, and *Klebsiella* in cecal microbiota. **C** The relative abundance of *Barnesiella*, *Bacteroides*, *Lachnoclostridium*, *Blautia*, and *Ruminococcus* in cecal microbiota. *P < 0.05, **P < 0.01. NC: negative control group; SP: positive control group; MccY: MccY-treated group.

The regulatory impact of MccY on the gut microbiota of chicks infected with *S*. Pullorum was analyzed by 16S rRNA high-throughput sequencing and bioinformatics, and a Venn diagram (Additional file 7) of the shared and unique OTUs indicated that 366 OTUs were shared among the three groups, the number of unique OTUs decreased in the order of NC group (2818) > SP group (2534) > MccY group (2191). Further analysis of the relative abundance of microbiota at the genus level was illustrated in Figure 9. The composition of intestinal flora changed significantly between different treatment groups. Compared with the NC group, *S.* Pullorum challenge increased (*P* < 0.05) (Figure 9B) the relative abundance of *Escherichia*, *Raoultella*, *Salmonella*, *Citrobacter* and *Klebsiella* by 37.93%, 6.75%, 3.21%, 3.01% and 1.49%, respectively, while significantly decreasing (*P* < 0.05) (Figure 9C) the relative abundance of *Bacteroides*, *Lachnoclostridium* and *Ruminococcus* by 26.69%, 1.96% and 0.40%. Notably, MccY treatment showed a reduction in the relative abundance of *Escherichia* by 39.31% (*P* < 0.05), *Raoultella* by 6.75% (*P* < 0.05), *Salmonella* by 3.20, *Citrobacter* by 2.96%, and *Klebsiella* by 1.38% (Figure 9B), but an increase in the relative abundance of *Barnesiella* by 11.05% (*P* < 0.05), *Blautia* by 1.75% (*P* < 0.05), *Bacteroides* by 17.70%, *Lachnoclostridium* by 1.77%, *Ruminococcus* by 0.31%, when compared with the SP group (Figure 9C). The results demonstrated that *S*. Pullorum colonized the intestines of chickens, causing an increase in the abundance of the *Salmonella* genus. Following treatment with MccY, 99.55% of *Salmonella* was effectively eliminated from the cecum, as evidenced by a decrease in its relative abundance from 3.2165% in the SP group to 0.0145% in the MccY group, indicating that MccY effectively mitigated the colonization of *Salmonella* in the gut compared to the SP group. MccY promotes intestinal ecological balance by reducing the relative abundance of harmful microbial communities, such as *Salmonella* and increasing the relative abundance of beneficial bacteria, in *S*. Pullorum-infected chicks.

## Discussion

Chickens are extremely vulnerable to *S*. pullorum infection during their early growth stages, leading to white diarrhea and a high mortality rate or asymptomatic persistent infection [24]. *Salmonella* Pullorum colonizes the cecum of chicks, destroys the intestinal mucosa, and invades the liver, causing lesions in the liver and intestinal tissue lesions, leading to weight loss in poultry [25]. Microcin MccY is a novel lasso peptide, which has high antibacterial activity against various *Salmonella* serotypes, including *S*. Pullorum [15]. In this study, the antibacterial activity of MccY against clinical isolates of *S*. Pullorum was determined. The results showed that MccY had high antibacterial activity against *S*. Pullorum isolates, with a MIC of 0.125 μM, among which the antibacterial activity against the standard strain CVCC 526 reached 2.5 μM. Consistent with the related studies on MccJ25, the antibacterial concentration of MccJ25 against the *S*. Enteritidis CMCC 50336, a standard strain of *Salmonella*, was 2 μg/mL, while it had a lower MIC value against clinical isolates [18]. Subsequently, an oral challenge method was employed by administering a bacterial solution containing 5 × 10^8^ CFU on days 3 and 5 to establish a Pullorum disease model, which was consistent with the symptom pattern of horizontal transmission infection in production [26]. After infection, all chickens exhibited symptoms of white diarrhea, and *S*. Pullorum was isolated from the feces for nearly two weeks, while a small amount of *S*. Pullorum was also found in the liver and cecum without causing systemic infection or acute mortality. In other studies, injection of *S*. Pullorum CCVC 526 into 22-week-old chickens resulted in its detection in the liver, spleen, and cecum; however, this observation was not made in our experiment which may be attributed to the oral challenge method employed here [27], but it was not observed in this experiment, which may be related to the oral challenge method selected in this experiment. Different invasion methods of the strain affect its colonization in chickens and produce different infection results. In summary, the clinical *S*. Pullorum isolate 429 was selected in this study to construct a naturally infected chicken model of Pullorum disease, which helped explore the antibacterial effect of MccY in livestock production.

Due to the recent discovery of MccY, there has been a lack of research on its application in poultry. Similarly, MccJ25, which was identified in *E. coli*, has been utilized as a feed additive in broiler production due to its potent antibacterial activity against most strains of *E. coli* and certain *Salmonella*, as well as its robust resistance to heat and acid [13]. However, it should be noted that Yu et al. observed intestinal inflammation in mice following prolonged exposure to high doses of MccJ25 [28]; thus, caution must be exercised when implementing MccJ25 within safe thresholds. Consequently, prior evaluation of the safety profile of MccY in vivo is imperative before considering its application in poultry.

Following a two-week administration of different doses of MccY and PBS, weight monitoring results revealed no significant differences in body weight and daily gain between the 5 mg/kg, 10 mg/kg, and 20 mg/kg MccY groups compared to the control group. Notably, at day 19, the body weight of the 20 mg/kg MccY group exhibited an upward trend when compared with the control group. These findings align with a previous study on MccC7 chicks which reported similar results; no difference in daily gain was observed during the breeding stage (1–21 days old) upon the addition of MccC7 to their feed. However, during the fattening stage (21–42 days old), daily gain significantly surpassed that of the control group while also exhibiting a lower feed-to-gain ratio [29]. The results demonstrated that the administration of 20 mg/kg MccY did not exert any detrimental impact on the growth of chicks, and there were no discernible disparities in serum IgA and IgM levels across all experimental groups. These findings align with previous observations regarding the application of MccC7. Intestinal microbial analysis revealed that oral administration of 10 mg/kg and 20 mg/kg MccY effectively enhanced the chicken intestinal flora and increased the relative abundance of probiotics. Therefore, the optimal dosage range is between 10 and 20 mg/kg for clinical application. However, it should be noted that this study primarily focused on the safety evaluation of MccY with a relatively short test duration, thus failing to include monitoring of production performance such as feed-to-meat ratio and feed conversion rate in chickens. This limitation highlights the need for simulated clinical trials at different stages of growth to validate the impact on production performance if MccY is developed as an animal growth-promoting product. Intestinal microbial analysis revealed that oral administration of 10 mg/kg and 20 mg/kg MccY effectively enhanced the chicken intestinal flora and increased the relative abundance of probiotics. Therefore, for clinical application, the optimal dosage range is between 10 and 20 mg/kg. However, it should be noted that this study primarily focused on the safety evaluation of MccY with a relatively short test duration, thus failing to include monitoring of production performance such as feed-to-meat ratio and feed conversion rate in chickens. This limitation highlights the need for simulated clinical trials at different stages of growth to validate the impact on production performance if MccY is developed as an animal growth-promoting product.

In the present study, chicks challenged with *S*. Pullorum 429 exhibited depression, lethargy, poor growth, and white diarrhea, which was consistent with a previous study [2]. However, MccY treatment reinstated the BW and ADG of chicks to the level of the NC group, which is similar to former studies where MccJ25 alleviated weight loss caused by intestinal pathogenic bacterial infections [13].

When chicks are infected with *S*. Pullorum, this bacterium colonizes the intestine and spreads horizontally through fecal excretions [26]. This study evaluated the sterilizing efficacy of MccY against *S*. Pullorum in chickens by determining the quantity of *S*. Pullorum in feces, liver and cecal tissues. The results showed that MccY significantly reduced the *S*. Pullorum load in feces, liver and cecal tissue. Although *S*. pullorum was successfully colonized in the intestines of chicks during this experiment, it was difficult to monitor the bacterial load in the feces after two weeks of infection. This was related to the fact that after 3–5 days of intestinal colonization, the fecal load of *S*. pullorum gradually decreased after reaching its peak, making it difficult to monitor [26]. Interestingly, *S*. Pullorum was still present in the cecum at lower concentrations, although it was not detected in the feces of the MccY-treated group at dpi 14 and 21. This could be attributed to the limited ability of MccY to eradicate *S.* Pullorum from intestinal cells, as it was constrained by its inability to traverse the gut barrier and instead remained confined to the intestinal surface, exerting its bactericidal function. Similarly, it is hypothesized that MccY cannot effectively cross the blood barrier to fully eliminate Salmonella from the liver. Thus, studies on the prevention of *S*. Pullorum infections by MccY have been consequently taken into consideration to eradicate the bacteria in the earliest phases of the pathogen infection and achieve better treatment. In conclusion, MccY was able to resist *S*. Pullorum infection in chickens, thereby alleviating gut invasion, reducing intestinal colonization, and decreasing the possibility of horizontal transmission in chicks.

The immune organ index is an important measure of immunological health in poultry [30]. The weights of the thymus and bursa of Fabricius are used to assess the immunological status of chickens, and the larger the proportional weight of the organs is, the stronger the cellular and humoral immunity [31]. The bursa of Fabricius, a unique immune organ, helps chicks develop B lymphocytes, which are essential for the adaptive immune response [32]. *Salmonella* infection causes changes in immune organs; for example, the thymus volume decreases when *S*. Typhimurium invades the thymus [33]. In the present study, the thymus index showed a downward trend during the period of *S*. Pullorum infection, indicating that *Salmonella* infection harmed thymus development, whereas treatment with MccY restored the volume of the thymus and the bursa of Fabricius to preinfection levels, suggesting that MccY improved immune function in the chicks infected with *S*. Pullorum. This finding was similar to another study in which the probiotic *Lactobacillus* was used to advance the growth and development of the thymus and bursa of Fabricius to alleviate *S*. Pullorum infection in chickens [34, 35].

The intestinal barrier is the first line of defense against invading pathogens, and the integrity of the intestinal epithelium is important for the digestive, absorptive, and protective functions of the intestine, as well as serving as a barrier to prevent pathogens and their products from entering the body [36]. *S*. Pullorum attacks the intestinal mucosa of chicks and produces toxins, causing damage to the intestinal mucosal barrier. Moreover, it caused an increase in the crypt depth of the intestinal villus and a decrease in the V/C, which is responsible for weight loss and low feed conversion in chicks [25]. In this study, after infection with *S*. Pullorum, there was inflammatory damage to the jejunal and ileal tissues in the SP group, along with a significant increase in the jejunal and ileal crypt depth and a statistical decrease in the V/C compared to the NC group. This impaired intestinal morphology reduced the absorptive area of the small intestine and decreased the ability to absorb nutrients, which might have contributed to the significantly lower body weight in the SP group. In contrast, compared to the SP group, oral administration of MccY increased the jejunal villus height, decreased the jejunal and ileal crypt depth, and returned the V/C to the level of the NC group. This finding was comparable to earlier microcin studies, where feeding MccC7 raised the villus heights of the duodenum and jejunum, reduced the crypt depths of the duodenum and ileum crypt depths, and increased the V/C of the duodenum, jejunum, and ileum [29].

Cytokines are vital for the regulation of the immune system, and anti-inflammatory cytokines, such as IL-4 and IL-10 can hinder the development of inflammatory responses [37]. A previous study reported that adding MccJ25 to the diet significantly increased the cytokine IL-10 in the serum of weaned piglets [12]. In this study, MccY was found to upregulate the mRNA expression of *IL-4* and *IL-10* in chicks compared to the SP group, which may be beneficial in reducing the negative effects of* S*. Pullorum infection in chickens.

The tight junctions between intestinal epithelial cells, consisting of adhesion molecules, the transmembrane proteins Occludin, the Claudin family, and connexins, which can regulate intestinal permeability, are an essential part of the gut barrier. Disruption of tight junctions results in intestinal inflammation, tissue injury, and nutrient loss [38]. Additionally, intestinal mucus, which contains mainly Mucin-2, is another important part of the gut barrier that covers the surface of the intestinal epithelium, keeps the intestinal surface clean, and resists pathogens [39]. Similar to the upregulation effect of MccC7 [29], MccY significantly upregulated the mRNA expression of *ZO-1* in the jejunum and ileum and *CLDN-1* in the jejunum compared to the SP group, indicating that MccY may enhance intestinal tight junctions and improve the intestinal barrier to resist *S*. Pullorum infection.

The intestinal microbiota, known as the “invisible organ”, can enhance intestinal feed digestion and nutrient absorption, improve animal growth performance, promote body health, and strengthen intestinal barrier function [40]. The imbalance of the gut microbiota can trigger metabolic, autoimmune, and inflammatory diseases, and disrupt intestinal function [41]. For this reason, helping chicks establish a stable gut flora is crucial to preventing intestinal pathogenic bacterial infections. This study further investigated the relative abundance of the gut microbiome at the genus level. In this study, chicks infected with *S*. Pullorum reduced the relative abundance of *Bacteroides*, *Lachnoclostridium* and *Ruminococcus*, as well as increased the relative abundance of *Escherichia*, *Raoultella*, *Citrobacter*, *Salmonella* and *Klebsiella*. The decreased abundance of *Bacteroides* and *Lachnoclostridium*, which are related to the nutrient absorption and decomposition abilities of poultry, could be one of the reasons for weight loss in the SP group. A previous study found that microcin primarily resides in the animal gut and maintains the balance of the gut microbiota by increasing the probiotic flora and inhibiting intestinal pathogens [42]. In this study, the addition of MccY significantly reduced the relative abundance of *Escherichia* and *Raoultella*. The relative abundance of *Salmonella* in the SP group was 3.2165%, whereas it decreased to 0.0145% in the MccY group, demonstrating an elimination rate of 99.55% for *Salmonella* within the intestinal microbiota, which aligns with previous findings on fecal and cecal bacterial loads. This indicated that MccY inhibited the colonization of *Salmonella* and had a promising therapeutic effect on infected chicks. Li et al. discovered that using antibiotics infrequently and at high doses caused some bacteria to become resistant, which disrupts the intestinal ecology [43]. In contrast, following MccY therapy, the structure of the gut microbiota was closer to that of the NC group, as evidenced by a decline in the relative richness of pathogenic bacteria and an increase in the relative abundance of probiotics, which mitigated the dysbiosis induced by *S*. Pullorum infection and improved the health of the intestinal flora in chicks. Further studies on anti-inflammatory mechanisms and modifications in bacterial metabolism are needed to better understand the mechanism of MccY.

### Supplementary Information

**Additional file 1**. **Strains used in this study.**

**Additional file 2**. **Plasmids used in this study.**

**Additional file 3**. **Primers used in this study.**

**Additional file 4**. **Sensitivity of S. Pullorum to MccY and MccJ25.**

**Additional file 5**. ***S.*** **Pullorum load in feces, liver, and cecum. A** *S*. Pullorum load in feces. **B** *S*. Pullorum load in liver. **C** *S*. Pullorum load in cecum. Dates were shown as the means ± SEM (*n* = 3). dpi: days post-infection.

**Additional file 6**. **Effects of oral administration of MccY on the α-diversity indices of cecal microbiota at day 19**. **A** Venn diagram showing the shared and unique OTUs in different groups. **B** Simpson index, Simpson index, Chao index and Observed species.

**Additional file 7**. **Venn diagram showing the shared and unique OTUs in chicks infected with** ***S.*** **Pullorum.**

## Data Availability

The datasets used and/or analyzed during the current study are available from the corresponding author upon reasonable request.

## Abbreviations

ADG: average daily gain
ANOVA: analysis of variance
BW: body weight
CD: crypt depth
CLDN-1: Claudin-1
CFU: colony forming unit
dpi: days post-infection
IFN-γ: interferon-γ
IL-4: interleukin-4
IL-6: interleukin-6
IL-10: interleukin-10
LEfSe: linear discriminant analysis effect size
MUC-2: Mucin-2
OCLN: Occludin
OTUs: operational taxonomic units
PBS: phosphate buffer saline
PCoA: principal coordinate analysis
SEM: standard error of mean
TNF-α: tumor necrosis factor-α
VH: Villus height
V/C: the ratio of villus height to crypt depth
ZO-1: zonula occludin-1
