# Supplementary material for: Effect of oral administration of microcin Y on growth performance, intestinal barrier function and gut microbiota of chicks challenged with Salmonella Pullorum
Source: Vet Res. 2024 May 22;55:66. doi: 10.1186/s13567-024-01321-x (PMC11112776; doi:10.1186/s13567-024-01321-x)
Supplement: Supplementary file 1 — Additional file 1. Strains used in this study. [file 13567_2024_1321_MOESM1_ESM.docx]

**Additional file 1. Strains used in this study.**

| Name | Relevant characteristic(s) | Source |
| --- | --- | --- |
| 284 | *Salmonella Pullorum* isolate, wild type | Laboratory collection |
| 429 | *Salmonella Pullorum* isolate, wild type | Laboratory collection |
| 431 | *Salmonella Pullorum* isolate, wild type | Laboratory collection |
| CVCC 526 | *Salmonella Pullorum* CVCC 526, wild type | Laboratory collection |
| DH5α | F^-^, φ80d/*lacZ*ΔM15, Δ(*lacZYA*-*argF*) U169 *recA*1 *endA*1 *hsdR*17 | Laboratory collection |
| BL21 | F^-^, *ompT hsdS_B_* (r_B_^-^, m_B_^-^) *gal dcm* (DE3) | Laboratory collection |
| YL01 | pYL01 in *Escherichia coli* BL21 (F^-^, *ompT hsdSB* (r_B_-, m_B_-) *gal dcm* (DE3)) | [15] |
| YL02 | pYL02 in *Escherichia coli* BL21 (F^-^, *ompT hsdSB* (r_B_-, m_B_-) *gal dcm* (DE3)) | [15] |
