# Supplementary material for: Effect of oral administration of microcin Y on growth performance, intestinal barrier function and gut microbiota of chicks challenged with Salmonella Pullorum
Source: Vet Res. 2024 May 22;55:66. doi: 10.1186/s13567-024-01321-x (PMC11112776; doi:10.1186/s13567-024-01321-x)
Supplement: Supplementary file 2 — Additional file 2. Plasmids used in this study. [file 13567_2024_1321_MOESM2_ESM.docx]

**Additional file 2. Plasmids used in this study.**

| Name | Relevant characteristic(s) | Source |
| --- | --- | --- |
| pYL01 | *mcyABCD* gene cloned in pET28 (a) with BamHI and SalI, Kan^R^ | [15] |
| pYL02 | *mcjABCD* gene cloned in pET28 (a) with BamHI and SalI, Kan^R^ | [15] |
