# Supplementary material for: Effect of oral administration of microcin Y on growth performance, intestinal barrier function and gut microbiota of chicks challenged with Salmonella Pullorum
Source: Vet Res. 2024 May 22;55:66. doi: 10.1186/s13567-024-01321-x (PMC11112776; doi:10.1186/s13567-024-01321-x)
Supplement: Supplementary file 3 — Additional file 3. Primers used in this study. [file 13567_2024_1321_MOESM3_ESM.docx]

**Additional file 3. Primers used in this study.**

| Primer | Sequence (5' to 3') |
| --- | --- |
| *GAPDH* | F: AACTTTGGCATTGTGGAGGG |
|  | R: ACGCTGGGATGATGTTCTGG |
| *IL-4* | F: GTGCCCACGCTGTGCTTAC |
|  | R: AGGAAACCTCTCCCTGGATGTC |
| *IFN-γ* | F: CCTCGCAACCTTCACCTCAC |
|  | R: CGCTGTAATCGTTGTCTTGGAG |
| *IL-6* | F: AATGCCTGACGAAGCTCTCC |
|  | R: CTCGACGTTCTGCTTTTCGC |
| *IL-10* | F: TTTGGCTGCCAGTCTGTGTC |
|  | R: TGATGACTGGTGCTGGTCTG |
| *TNF-α* | F: CCGCCCAGTTCAGATGAGTT |
|  | R: GCAACAACCAGCTATGCACC |
| *ZO-1* | F: CTTCAGGTGTTTCTCTTCCTCCTC |
|  | R: CTGTGGTTTCATGGCTGGATC |
| *CLDN-1* | F: CATACTCCTGGGTCTGGTTGGT |
|  | R: GACAGCCATCCGCATCTTCT |
| *OCLN* | F: ACGGCAGCACCTACCTCAA |
|  | R: GGGCGAAGAAGCAGATGAG |
| *MUC-2* | F: TTCATGATGCCTGCTCTTGTG |
|  | R: CCTGAGCCTTGGTACATTCTTGT |
| 16s-338F | F: ACTCCTACGGGAGGCAGCA |
| 16s-806R | R: GGACTACHVGGGTWTCTAAT |
