# Supplementary figures and images for: Effect of oral administration of microcin Y on growth performance, intestinal barrier function and gut microbiota of chicks challenged with Salmonella Pullorum
Source: Vet Res. 2024 May 22;55:66. doi: 10.1186/s13567-024-01321-x (PMC11112776; doi:10.1186/s13567-024-01321-x)

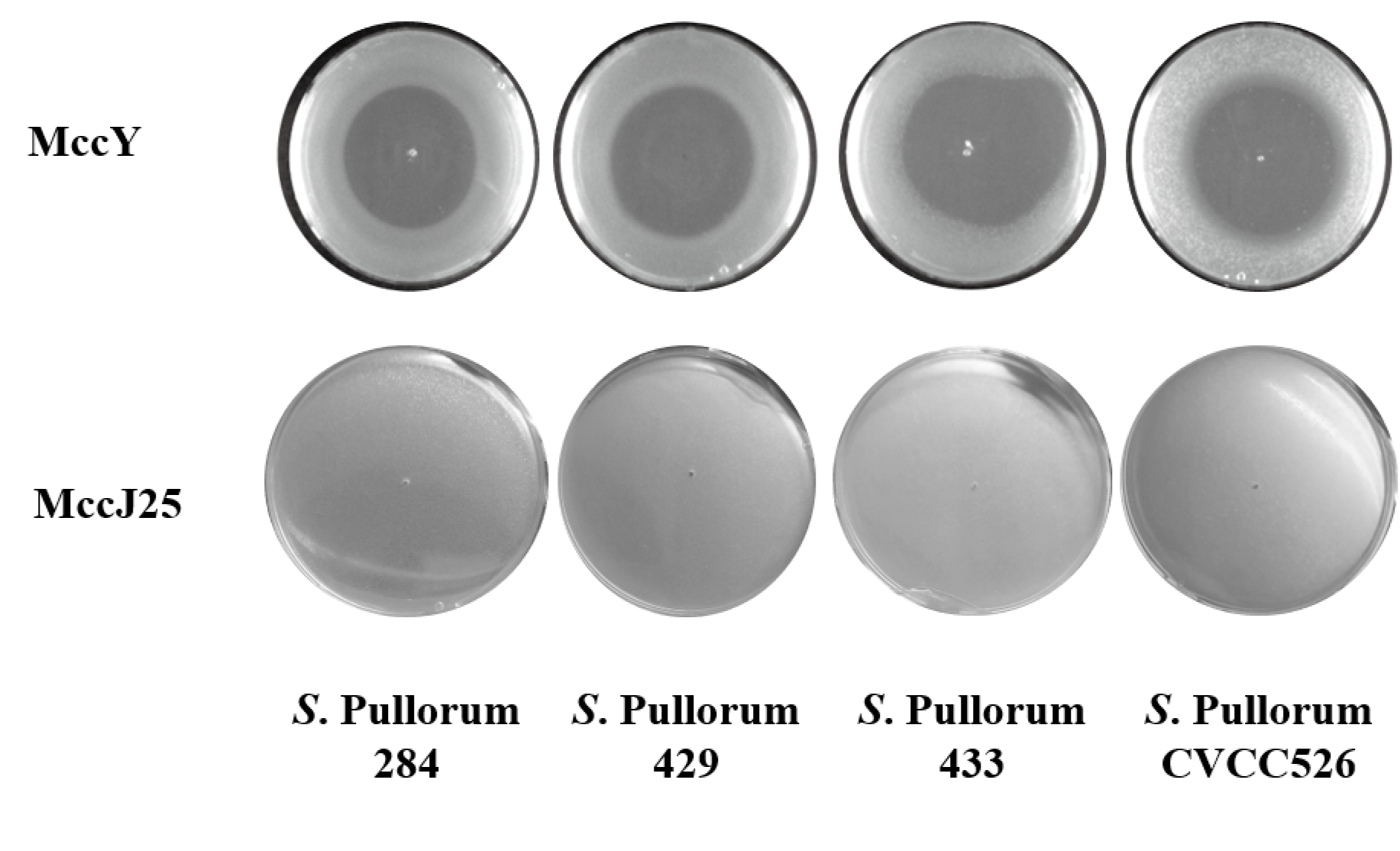

Supplement: Supplementary file 4 — Additional file 4. Sensitivity of S. Pullorum to MccY and MccJ25. [file 13567_2024_1321_MOESM4_ESM.tif]

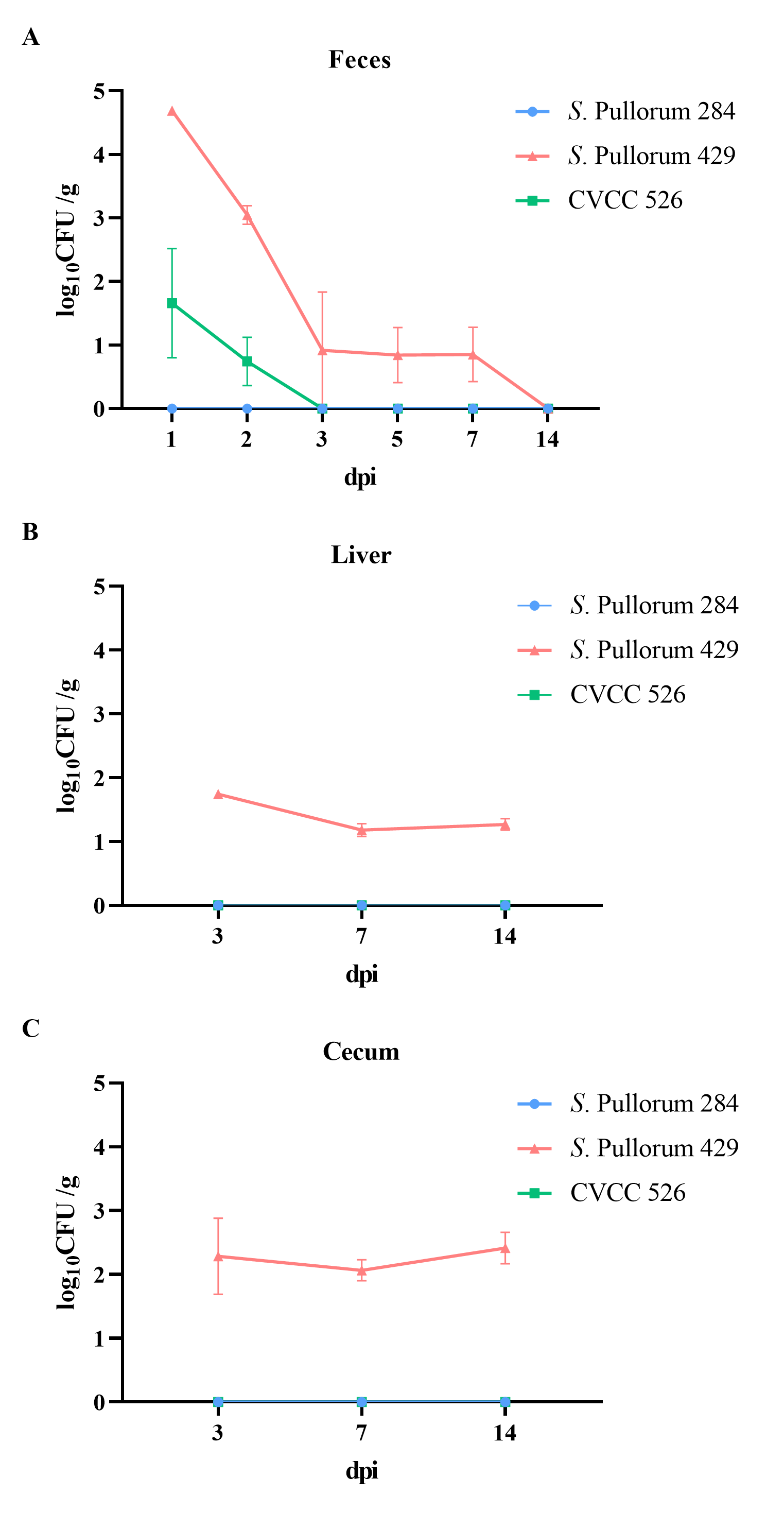

Supplement: Supplementary file 5 — Additional file 5. S. Pullorum load in feces, liver, and cecum. A S. Pullorum load in feces. B S. Pullorum load in liver. C S. Pullorum load in cecum. Dates were shown as the means ± SEM (n = 3). dpi: days post-infection. [file 13567_2024_1321_MOESM5_ESM.tif]

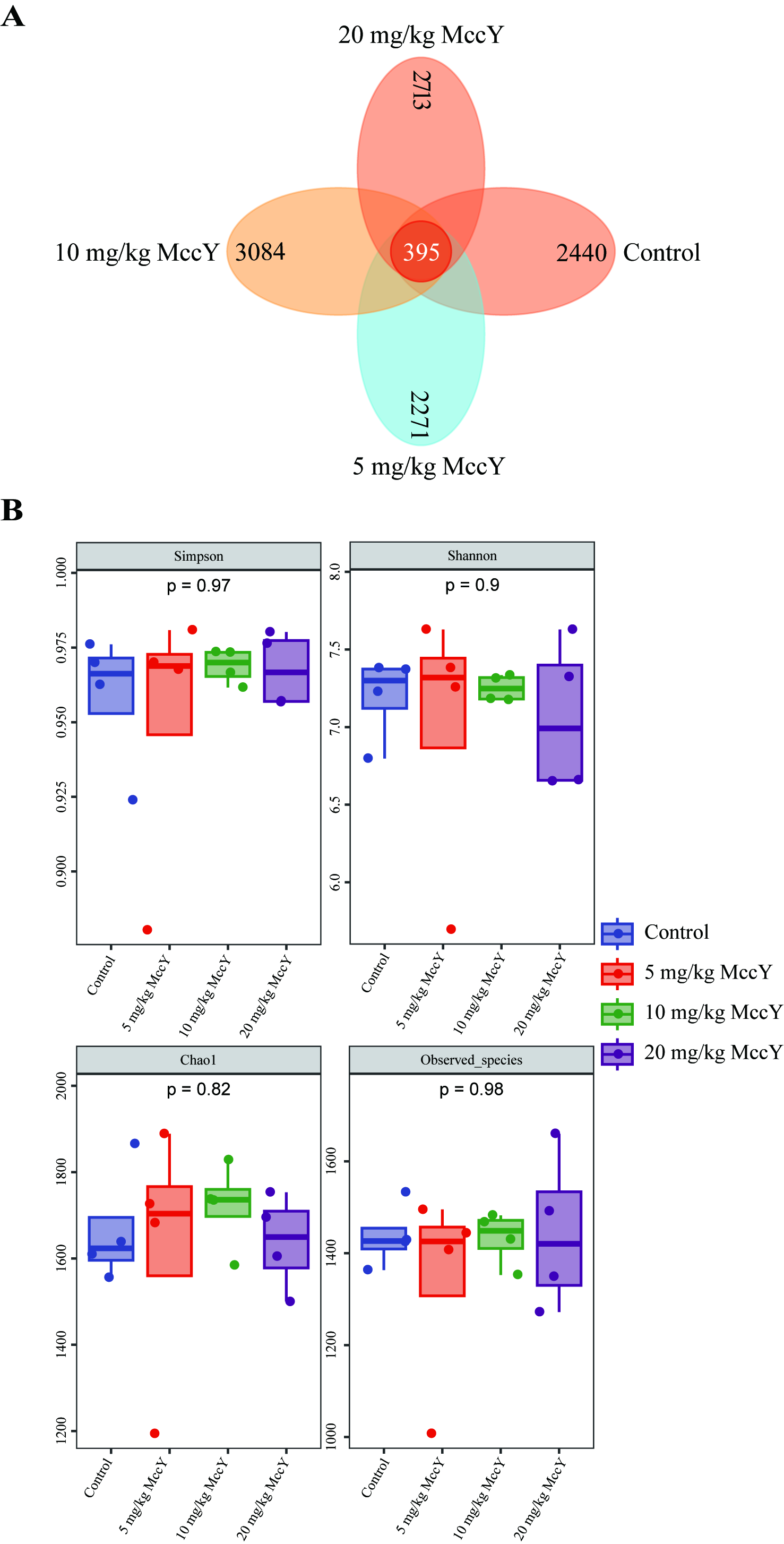

Supplement: Supplementary file 6 — Additional file 6. Effects of oral administration of MccY on the α-diversity indices of cecal microbiota at day 19. A Venn diagram showing the shared and unique OTUs in different groups. B Simpson index, Simpson index, Chao index and Observed species. [file 13567_2024_1321_MOESM6_ESM.tif]

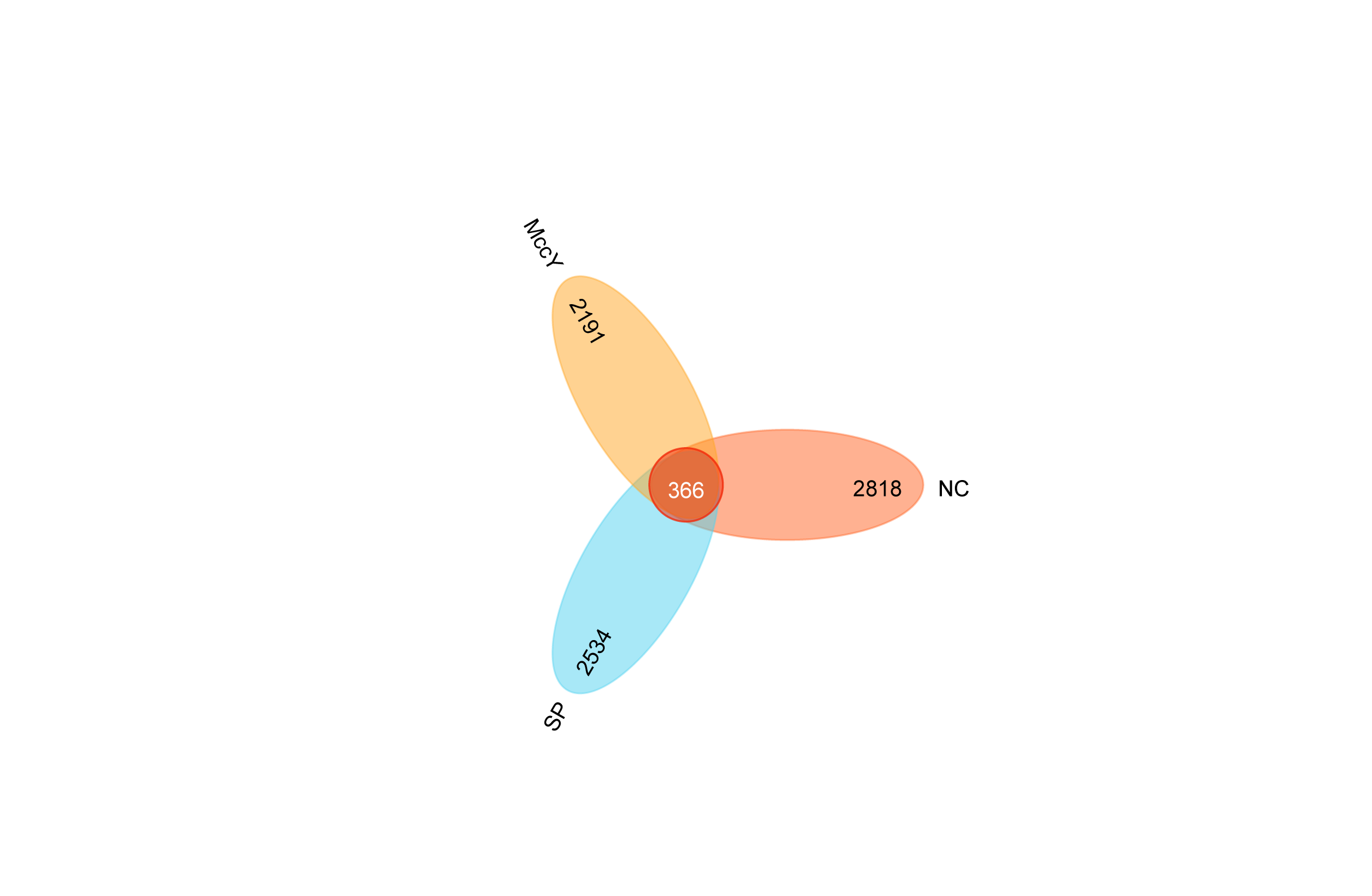

Supplement: Supplementary file 7 — Additional file 7. Venn diagram showing the shared and unique OTUs in chicks infected with S. Pullorum. [file 13567_2024_1321_MOESM7_ESM.tif]
